# Effect of fasting and two different photoperiods on immune parameters in adult male and female house crickets (*Acheta domesticus*)

**DOI:** 10.1016/j.cirep.2025.200210

**Published:** 2025-02-07

**Authors:** Frida A. Lindberg, Ida Waern, Emma Nilsson, Anna Jansson, Lena Holm, Erika Roman

**Affiliations:** a Department of Animal Biosciences, Swedish University of Agricultural Sciences, Box 7023, Uppsala 750 07, Sweden; b Department of Pharmaceutical Biosciences, Uppsala University, Box 591, Uppsala 751 24, Sweden

**Keywords:** Acheta domesticus, Cricket rearing, Starvation, Immunocompetence, Welfare

## Abstract

Insects are often reared as a potential source of protein, both for human and animal consumption. However, food safety is a main concern and one common way of reducing the microbial load in insects is fasting them for 24–48 h. Fasting may pose a physiological stress with potential implications for welfare. Another parameter that can be manipulated in insect rearing facilities is the photoperiod, which affects maturation of the insects, and possibly immunity. In this study, adult *Acheta domesticus* of both sexes, reared in either 16 (16L:8D) or 12 h (12L:12D) of light, were fasted for 48 h or fed *ad libitum* prior to euthanasia. The immune parameters phenoloxidase (PO) activity, lysozyme concentration, haemocyte number and total protein content were investigated in haemolymph. The results revealed effects of both photoperiod and fasting on immunocompetence. Fasting lowered PO activity (*p* = 0.002) and haemocyte count (*p* = 0.005). The haemocyte count was especially affected by fasting in female crickets, and haemocyte count was correlated to protein concentration, and to PO activity in crickets reared in 16L:8D. A 16L:8D photoperiod lowered the protein content in haemolymph compared to 12L:12D (*p* = 0.045). Protein content has been correlated to disease resistance in previous studies, and was herein found to be correlated with PO activity. These results need to be replicated and investigated further to make recommendations for future cricket rearing, but the results point to the fact that rearing practices could have consequences for insect immunity.

## Highlights

• *A. domesticus* reared in two different photoperiods were fasted for 48 h.
• Haemolymph parameters phenoloxidase, lysozyme, haemocytes and protein were measured.
• Both fasting and photoperiod affected haemolymph immune parameters.
• Most affected was the haemocyte count (decreased) in fasted females.
• Rearing practices could have effects on immune function, indicating welfare issues.

## Graphical abstract

**Figure:**
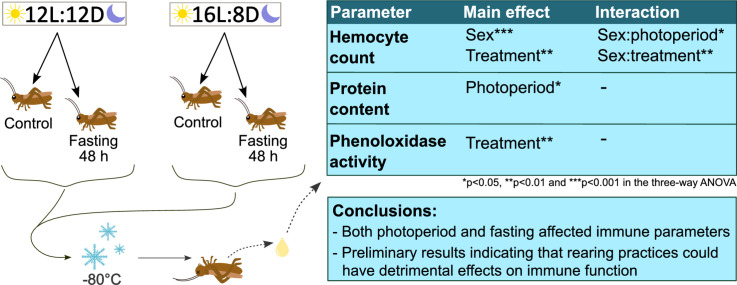

## Introduction

Insects have, in large parts of the world, been part of the diet and consumed by humans for thousands of years, and have been proposed as an alternative food source for an increasing world population [[1], [2], [3]]. The house cricket *Acheta domesticus* (Orthoptera, Ensifera) is often reared for human consumption due to the simplicity of rearing and nutritional value. High protein content, unsaturated fats and micronutrients, such as calcium, iron and several vitamins (reviewed by [3]), makes *A. domesticus* a nutritious insect and a promising alternative to traditional protein sources.

Growth and maturation of *A. domesticus* into adults is known to be affected by temperature [4] and photoperiod [5], hence is rearing environment a crucial factor to consider. Temperature is important for insects to hatch, grow and develop, and to reproduce [4,[6], [7], [8], [9], [10]]. The impact of photoperiod has been investigated in several species and seems to be especially important for univoltine insects [[5], [6], [7],^9^]. In crickets, time to maturity and weight varies considerably with photoperiod [11,12]. According to Clifford and Woodring [4], *A. domesticus* reaches final moult after 45 days using a 14L/10D (L, hours of light; D, hours of dark) photoperiod in 30 °C, but the ideal photoperiod for rearing has been less studied. For instance, the Food and Agricultural Organization of the United Nations (FAO) recommends rearing temperature for crickets, but does not mention a recommended photoperiod to be used [13]. Generally a longer maturation time results in larger individuals, e.g. in *Gryllus vocalis, Teleogryllus emma, Modicogryllus siamensis* and Gryllus *bimaculatus* [9,11,14,15]. This may in turn have effects on other traits such as the immune system [15]. There are studies indicating that photoperiod affects immunity in other insects [[16], [17], [18]]. Due to the energetic investment in immunity, it is likely that when growth and development are affected by altered temperature and/or photoperiod, a trade-off on immunity is made in *A. domesticus*, too.

Edible insects, including *A. domesticus*, are often eaten whole, either directly or ground into flour. Due to the high microbial load in the gut there could be a potential risk for human consumption [19]. It has therefore been suggested to fast insects 24–48 h prior to euthanasia to empty the gut [e.g. 20]. However, recent studies have shown that fasting has no effect on microbial load in *A. domesticus* [21]. Rather, fasting affects the behaviour of insects [22], e.g. increased shelter use in *A. domesticus* [23], indicating possible compromised welfare. Moreover, the insect's immune system is known to be affected by physiological stressors, including fasting [24,25]. Starvation increases mortality in bumblebees when presented with an immune challenge [26], indicative of the importance of proper energy access. Thus, fasting may pose a potential risk of a decreased disease resistance, leading to population loss and affecting financial profit for the breeder, alongside the health and welfare problem.

Although it is complicated to measure disease resistance alone [27], measuring important parameters within the insect immune system could give insights into its immunocompetence. Common immune parameters to measure in haemolymph are lysozyme and phenoloxidase (PO) activity, and haemocyte count, as they are important parts of insects’ humoral and cellular immune system [8,17,[27], [28], [29]]. Lysozyme is constantly present in the haemolymph and is a common measure of antimicrobial immunity. It is one of the most important antimicrobial defences in Orthoptera due to their lack of antimicrobial peptides [30].

PO activity and haemocyte count have been associated with survival of bacterial challenge in *G. bimaculatus* [8], and are therefore often used as measures of immunocompetence. Haemocytes and PO are produced constantly, and their function is therefore costly for the animal. If an insect is challenged, e.g. by fasting, it is likely that these parameters are affected.

Although not a commonly used immune parameter, the total protein content in the haemolymph has previously been correlated to disease resistance [27], and is another parameter that is likely to be affected by fasting, hence a parameter that could be of importance for immunocompetence as well.

Taken together, it is important to investigate the impact of rearing practices, especially the fasting that occurs before euthanasia, on immune function since it could have implications for insect health. The current study, therefore, aims to elucidate the effects of fasting on the immune parameters PO activity, lysozyme concentration, haemocyte number, and total protein content in male and female *A. domesticus* reared in different photoperiods.

## Material and methods

### Animals and housing

An outbred population of laboratory-reared *A. domesticus* regularly backcrossed with wild-caught crickets from Sweden [31] was used. The wild-caught crickets were tested negative for the *A. domesticus* densovirus [32] before being introduced into the laboratory-reared population.

The crickets had been reared in 16 × 13 × 13 cm transparent boxes for three weeks and then moved to 28 × 20 × 28 cm boxes until maturity. All boxes had steel nets on one side for ventilation and were equipped with black plastic tubes as shelter [33], *ad libitum* wheat- and oat-based pellet feed [31], water (10 ml cotton plugged tubes), and salt [34]. The boxes were held in two separate cabinets with glass shelves and string lights mounted at three different levels (lights on at 07:00 CET). The temperature was kept at 30 ± 1 °C and relative humidity at 45–55 %. The crickets used in this study were 101–102 days old from hatching.

### Experimental design

Mature *A. domesticus,* reared for a different purpose in either 16L:8D or 12L:12D (L, hours of light; D, hours of dark) photoperiod (from egg until euthanasia), were used. From five replicates in each photoperiod, two females and two males from each replicate were subjected to the treatments: fasting for 48 h (fasted), or fed *ad libitum* (control) (except for one less female reared in 16L:8D and four less males in 12L:12D – due to not enough crickets of each sex per replicate). In total, 75 crickets were included in the study: 39 crickets reared in 16L:8D (19 females (9 fasted, 10 control), 20 males (10 per treatment)) and 36 crickets reared in 12L:12D (20 females (10 per treatment), 16 males (9 fasted, 7 control)). Crickets from the same replicate were housed together. Four animals were found to have escaped (one broken box; two females, two males) and two females were found dead (both belonged to the control group). This gave rise to a final number of 69 crickets that were included in the study. A fasting period of 48 h was chosen over 24 h due to the behavioural effects previously observed [23]. Both experimental groups had *ad libitum* access to water. After 48 h, animals were weighed, marked with a colour on their pronotum and allowed to rest for 30 min before euthanasia. Crickets were euthanized by putting the plastic containers with the crickets in −80 °C, where the animals were stored until further use.

### Haemolymph collection

Haemolymph was collected after euthanasia. *A. domesticus* were taken from the freezer and put on ice, legs were removed, and the coxa of the hind leg punctured with a dissection needle. The cricket was then allowed to thaw in room temperature until haemolymph extruded from either the punctuated coxa or through any of the removed legs. In total, 69 crickets were assayed, with successful haemolymph extraction from 65. One µl haemolymph was used for haemocyte count, the remaining haemolymph was diluted 1:25 in PBS (pH 7.4), vortexed for 5 s, aliquoted and stored in −80 °C until use in the PO, lysozyme and Bradford assays. A fresh aliquot was used for each assay. The assays were run within two months from collection date, all samples were run on the same plate, and each assay was run twice. The average result of both plates was used for analysis and a CV of < 15 % was used as a measure of acceptable variance between the two plates. Due to variation in extracted haemolymph volume (2–20 µl), not all crickets were represented in all assays.

### Haemocyte count

One µl haemolymph was diluted in 12.5 µl of anticoagulant buffer [98 mM NaOH, 146 mM NaCl, 16 mM EGTA, 10 mM citric acid, pH 6.5 [35]] and 1 µl of trypan blue. Ten µl of this mixture was then transferred to a haemocytometer chamber, and all non-stained haemocytes in three diagonal squares of the haemocytometer were counted under a microscope (Nikon Eclipse E200, Nikon Corporation, Japan). In total, haemocytes from 60 crickets were successfully counted.

### Phenoloxidase and lysozyme assays

Previous studies reported that measuring PO in cricket haemolymph did not result in detectable activity [27,36]. Hence a proPO activator (α-chymotrypsin) to transform proPO to the active PO enzyme was used. Total PO activity was thereby measured by a protocol modified from [27]. Briefly, 25 µl of diluted haemolymph (1:25 in PBS, pH 7.4) was mixed with 20 µl bovine α-chymotrypsin (2 mg/1.5 ml PBS; #C4129, Sigma-Aldrich, St. Louis, MO, US) in a 96-well plate and incubated at room temperature for 20 min. Seventy µl of l-Dopa (10 mM in PBS; #333,786, Sigma-Aldrich) was thereafter added to each well, and the absorbance was read at 490 nm every second minute for 60 min using an Infinite M1000 plate reader (Tecan Group Ltd, Switzerland). A negative control (no haemolymph) and a blank control (PBS only) were run on each plate. The linear phase (10–30 min) was used to calculate the change in absorbance per minute for each sample, and was used as a measure of total PO activity. In total, 60 crickets were assayed.

The protocol for measuring lysozyme concentration was modified from [27]. The diluted haemolymph was used, and 20 µl was added to a 96-well plate. A lysozyme standard (0.0016–0.0128 mg/ml, from chicken egg-white, #A3711, BioChemica, ITW Reagents, Spain) was added to the plate. *Micrococcus lysodeikticus* (500 µg/1.5 ml PBS, #M3770, Sigma-Aldrich) was thereafter added to the plate, which was directly read at 450 nm every second minute for 50 min (Tecan infinite M1000). The obtained results, i.e. change in absorbance per minute for each sample during the linear phase of the reaction (0–4 min) were corrected for activity in the negative control (no haemolymph). The standard curve was thereafter used to calculate amount of lysozyme in the samples. In total, 53 crickets were assayed.

### Protein measurement

Protein concentration in haemolymph was determined using a Bradford assay. Five µl of each sample (haemolymph diluted in PBS 1:25) and protein standard (bovine serum albumin, #5000207, Bio-Rad) were loaded onto a 96-well plate. Then 140 µl of Bradford reagent (#BA00050, OZ Biosciences) was added to sample, standard and negative (PBS instead of haemolymph) wells, and the absorbance read at 595 nm after 10 min (Tecan infinite M1000). In total, 62 crickets were assayed.

### Validation of haemolymph extraction method

Due to the large variation seen in extracted haemolymph volume, Spearman's rank correlation analysis was used to investigate whether this affected the measured parameters. No correlations were found between the collected haemolymph volume and the measured haemolymph parameters (haemocytes, *p* *=* *0.301*; PO, *p* *=* *0.214*; protein, *p* *=* *0.903*; and lysozyme*, p* *=* *0.348*), suggesting that the volume extracted did not influence the subsequent analyses. A moderate positive correlation was found between collected haemolymph volume and body weight (*r* = 0.4053, *p* *<* *0.001*), which is logic as a larger cricket has more haemolymph than a smaller cricket.

### Statistical analysis of data

Results from the PO, lysozyme and Bradford assays were checked in Grubb's outlier test (GraphPad QuickCalcs, Boston, MA, USA, http://www.graphpad.com/quickcalcs/grubbs1, accessed 15 January 2024), and a sample was excluded from the analysis if it was a significant outlier in the test, except if the outlier was one of three samples in the experimental group (one sample in the lysozyme assay). Duplicates with a CV-value of > 15 % were also omitted, except when absorbance values were close to zero (since small numbers require only small differences to affect the CV %). Due to the aforementioned reasons, one body weight measure, two haemocyte count samples, one sample from the PO assay, six samples from the Bradford assay, and one from the lysozyme assay were excluded from the analyses. Additionally, 19 samples were excluded from the lysozyme assay due to failure to reach the lowest activity measured by the standard curve. The final number of *A. domesticus* for each assay is summarized in Table 1.

**Table 1:** Final number of *A. domesticus* in each experimental group for the parameters studied.

	12L:12D (control)	12L:12D (fasted)	16L:8D (control)	16L:8D (fasted)
Haemocyte count	8 females, 7 males	8 females, 8 males	5 females, 7 males	6 females, 9 males
PO activity	9 females, 5 males	9 females, 7 males	6 females, 7 males	9 females, 7 males
Lysozyme concentration/activity	7 females, 3 males	6 females, 1 male	4 females, 3 males	6 females, 3 males
Protein content	8 females, 5 males	9 females, 6 males	5 females, 7 males	9 females, 7 males
Body weight	10 females, 6 males	10 females, 9 males	6 females, 8 males	9 females, 10 males

Control, *ad libitum* food; fasted, 48 h without feed; 12L:12D, 12 h of light and 12 h of dark; 16L:8D, 16 h of light and 8 h of dark; PO, phenoloxidase.

Data were analysed using Statistica 14.1 (TIBCO Software Inc., Tulsa, OK, United States) using a significance level of *p* *<* *0.05*. Generated data were analysed with a factorial analysis of variance (ANOVA), where photoperiod (16L:8D or 12L:12D), sex, and treatment (fasting or control) were set as independent factors and the measured variable as dependent. PO activity, lysozyme concentration and haemocyte count data were log-transformed to their respective natural logarithm to fulfil the normality assumption in the factorial ANOVA. The homoscedasticity was evaluated by normal probability plots. Scheffés post hoc analysis was used on statistically significant results from the ANOVA. Spearman's rank correlation was used to investigate if there were any overall correlations between the measured immune parameters and protein content, as well as in the different experimental groups. In the lysozyme assay, a large number of samples did not reach the lowest concentration in the standard curve (our threshold). Fischer exact test was therefore used to distinguish if there were any differences in the groups, i.e. if they reached the threshold of lysozyme activity or not (**Table S1**).

## Results

### Haemocyte count

**Fig. 1:**
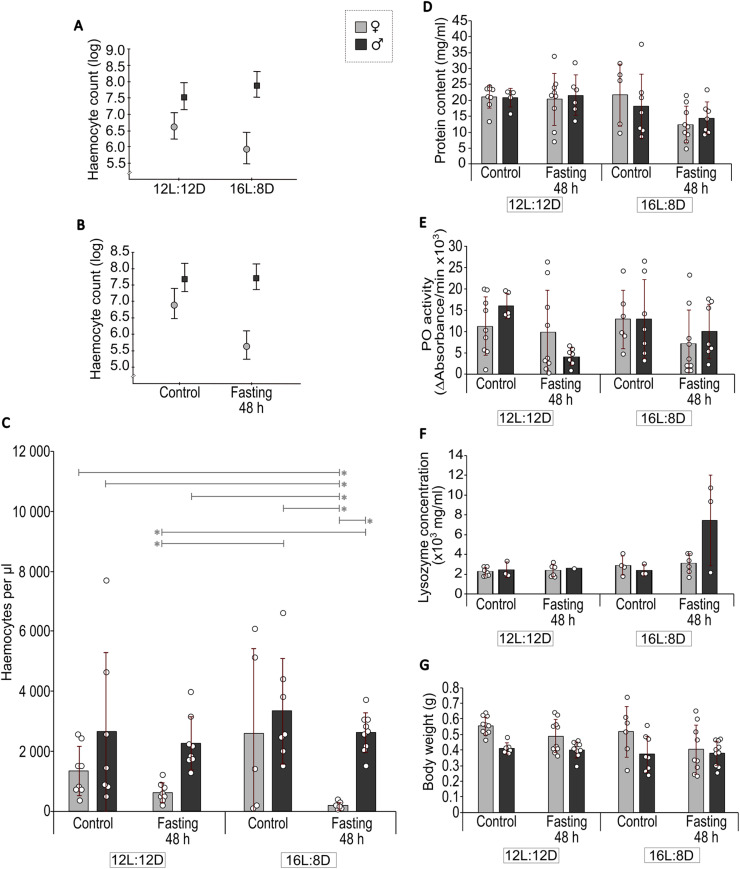
Effect of sex, treatment and photoperiod on immune parameters in haemolymph, and body weight from adult male and female *A. domesticus*. Number of circulating haemocytes (**A–C**, *n* = 5–9/group), protein content (**D**, *n* = 5–9/group), phenoloxidase (PO) activity (**E**, *n* = 5–9/group), lysozyme concentration (**F**, *n* = 1–7/group) in the haemolymph and body weight (**G**, *n* = 6–10/group) were measured. Interactions for haemocyte count were found in the three-way ANOVA between sex and photoperiod (**A**, Table 2) and sex and treatment (**B**, Table 2) respectively. Bars denote average values with standard deviation and each point corresponds to one individual. Vertical bars in **A** and **B** indicate 95 % confidence intervals. Analysis was made with a three-way ANOVA with Scheffés post hoc test. *p<0.05. 12L:12D, 12 h of light and 12 h of dark; 16L:8D, 16 h of light and 8 h of dark.

Haemocyte count is shown in Fig. 1**A-C**. There were main effects of treatment (fasted or not, *p* *=* *0.005*) and sex (*p* *<* *0.001*), and an interaction between photoperiod and sex (*p* *=* *0.018*), as well as between treatment and sex (*p* *=* *0.004*, Table 2). The interactions revealed that males tended to have an increased number of haemocytes in the haemolymph in the 16L:8D photoperiod than in 12L:12D, while the opposite pattern was seen in females (although not significant within sex; Fig. 1**A**). Moreover, circulating haemocytes tended to be lower in females during fasting, while no such effect was seen in males (Fig. 1**B**).

**Table 2:** Main effects and interactions from the three-way ANOVA of sex, photoperiod (12L:12D or 16L:8D) and treatment (*ad libitum* food or 48 h fasting) on haemolymph immune parameters and body weight in adult male and female *A. domesticus*.

Parameter	Main effect	Interaction
Haemocyte count	Sex*** Treatment** Photoperiod	Sex:Photoperiod* Sex:Treatment** Photoperiod:Treatment Sex:Photoperiod:Treatment
Protein content	Photoperiod* Treatment Sex	Sex:Photoperiod Sex:Treatment Photoperiod:Treatment Sex:Photoperiod:Treatment
PO activity	Treatment** Sex Photoperiod	Sex:Photoperiod Sex:Treatment Photoperiod:Treatment Sex:Photoperiod:Treatment
Lysozyme concentration	Photoperiod* Sex Treatment	Sex:Photoperiod Sex:Treatment Photoperiod:Treatment Sex:Photoperiod:Treatment
Body weight	Sex*** Photoperiod Treatment	Sex:Photoperiod Sex:Treatment Photoperiod:Treatment Sex:Photoperiod:Treatment

⁎ *p < 0.05*, **p < 0.01 and ***p < 0.001 in the three-way ANOVA.

Post hoc analyses showed that female crickets reared in the 16L:8D photoperiod and fasted for 48 h had a significantly lower number of circulating haemocytes than male crickets in all groups, and female control crickets reared in the 12L:12D photoperiod. Finally, fasted females in the 12L:12D photoperiod had a lower number of haemocytes than all males reared in 16L:8D (Fig. 1**C**).

### Protein content

Protein content is shown in Fig. 1**D**. There was a main effect (*p* *=* *0.045*) of photoperiod on protein concentration; crickets in the 16L:8D photoperiod had a lower haemolymph protein content than those in 12L:12D (Table 2).

Neither treatment (fasted or not) nor sex had any significant effects on protein content (Table 2), although there was a trend towards a lower amount of protein in haemolymph in fasted crickets (*p* *=* *0.06*). No interactions were found (Table 2), and the post hoc analysis revealed no differences between the experimental groups (Fig. 1**D**).

Protein content was positively correlated to PO activity in all crickets, in females, and in crickets reared in 12L:12D (**Table S2**). A positive correlation was also found with haemocyte count in general over all groups, in females, in fasted animals, and in subgroups of females (16L:8D, fasted; 12L:12D, control; **Table S2**).

### PO activity and lysozyme concentration

Phenoloxidase activity is shown in Fig. 1**E**. There was a main effect (*p* *=* *0.002*) of treatment on PO activity (Table 2), although no effects were seen in the post hoc analysis. No other effects in PO activity were found (Table 2, Fig. 1**E**). PO activity was positively correlated with haemocyte count in animals maintained in photoperiod 16L:8D (**Table S2**).

Lysozyme concentration is shown in Fig. 1**F**. Nineteen out of 53 samples had too low lysozyme concentration in the haemolymph i.e. did not reach the lowest concentration in the standard curve. More males than females failed to reach the threshold (30.2 % and 5.7 %, respectively; Fisher exact test, *p* *<* *0.001*; **Table S1**), suggesting that males in general had lower lysozyme concentration than females. Due to the high number of missing samples (**Table S1**), results from the remaining analysis should be interpreted with caution. There was a main effect (*p* *=* *0.038*) of photoperiod on lysozyme concentration (Table 2), but no differences were found in the post hoc analysis (Fig. 1**F**). The lysozyme-like activity (**Fig. S1**) followed the same pattern as the calculated lysozyme concentration (Fig. 1**F**).

### Body weight

Body weight is shown in Fig. 1**G**. Sex was the only parameter that affected body weight, with females as a group being heavier than males (Table 2, *p* *<* *0.001*). Neither photoperiod nor fasting had any significant effects on body weight (Fig. 1**G**) and there were no interactions (Table 2).

## Discussion

To the best of our knowledge, this is the first study investigating the effects of acute fasting on immune function in male and female *A. domesticus* reared in different photoperiods. The results show that both fasting and photoperiod affected the immune system, with pronounced effect of fasting on the number of haemocytes. Fasting also decreased PO activity, and photoperiod affected protein content and possibly lysozyme levels in haemolymph. These are important factors to consider in cricket rearing facilities, e.g. for food and feed and/or laboratory-reared colonies, where both different photoperiods and fasting of crickets are applicable.

### Haemocyte count, PO and lysozyme as measures of immunity

Fasting reduces the number of haemocytes for several species of insects [25], and a correlation between reduced number of haemocytes and decreased immunocompetence exists [25,37], which is why haemocyte count is often used as a measure of immunity. The results from the current study showed main effects of sex and fasting, and interaction effects between sex and treatment. Females, especially fasted females, showed lower haemocyte counts (Fig. 1**C**) compared to males that were unaffected (Fig. 1**B**). The finding of lower haemocyte count in females than males is in line with a study in *G. bimaculatus* [8], while sex differences were absent in other studies, e.g. in *Teleogryllus commodus* [14]. Moreover, the sexes were affected differently by photoperiod (Fig. 1**A**), but these results are more challenging to explain.

For PO activity in the current study, a main effect of treatment was found in line with previous findings [38], suggesting that energy intake is important for PO activity. Previous studies have also found that female *Gryllus texensis* and *T. commodus* of reproductive age, and female *G. bimaculatus* (3–5 days post adult emergence), have a higher PO activity than males of the same age [8,17,27,28]. However, this sex difference diminished as crickets got older [≥ 4 weeks post moult; [28]], which may explain the lack of sex difference in the current study using old *A. domesticus*.

Very low haemolymph lysozyme-like activity (**Fig. S1**) and concentration (Fig. 1**F**) were detected, in line with previous findings in *A. domesticus* [35]. More males than females failed to reach the threshold set for lysozyme concentration, suggesting that females could be more immunocompetent than males. Previous studies have reported decreased immunocompetence in males when reaching reproductive age [17,28]. However, another study found lysozyme activity to be higher in male *G. bimaculatus* than females [8]. Therefore, species-specific differences cannot be excluded. There was a main effect of photoperiod, possibly suggesting that longer days (16L:8D) result in higher levels of lysozyme in cricket haemolymph. However, when studying the data (Fig. 1**F**), the effect is driven by fasted males in the 16L:8D group, comprising only three individuals, and should therefore be interpreted with caution. Immunocompetence, measured as lytic activity and encapsulation rate, did not differ between sexes in *G. bimaculatus* (8 days post-eclosion; [15]), indicating uncertainties as to whether there are sex differences in parameters important for immunocompetence.

Taking the results from haemocyte count and PO activity into account, the effect of fasting is pronounced, suggesting a potential immunodeficit in these crickets. Females are possibly more sensitive to fasting than males, since females also produce eggs, another biological function that is costly, causing a trade-off between reproduction and immune function. Ecdysteroids, the steroid hormones in insects responsible for moulting processes, are also involved in reproductive function and can modulate innate immunity in response to stressors (reviewed by [39]). These hormones are likely to be affected by the nutrient deficiency (fasting treatment) and could thereby be one explanation to the effects seen in females.

### Haemolymph protein content and body weight

No sex differences were found in protein content, which is in line with previous results [40]. There was a main effect of photoperiod but no differences between experimental groups in the post hoc test (Table 2, Fig. 1**D**). Fasting for 48 h resulted in a trend (*p* *=* *0.06*) towards lower protein content, which points in the same direction as previous work showing that protein content was affected by food limitation in *G. texensis* [38]. Total amount of protein in the haemolymph is not by itself an immune parameter, but has been correlated with disease resistance to different types of bacteria [27], possibly due to a higher concentration of proteins important for immune function. These results suggest that the photoperiod used during rearing could have implications for haemolymph protein content, which might affect immunity as well as other bodily functions.

Cricket body weight was unaffected by 48 h of fasting. However, the crickets still had access to water, and a previous study showed that 48-h fasted *A. domesticus* (last instar) larvae actually increased their body weight [41]. Moreover, the increase in body weight was due to an increased plasma volume from increased water intake, without altering plasma protein content [41], which is in line with our results on adult *A. domesticus* (Table 2, Fig. 1**D and G**). Although no data on weight before fasting were obtained, this could explain why no differences on either body weight or protein content between the treatment conditions were found. This could also explain the lower concentration of haemocytes found in the fasted crickets, i.e. increased plasma volume but the same number of cells (Table 2, Fig. 1**C**). However, as discussed by Woodring [41], the increase in water intake in last instar larvae could have been to ensure that moult would happen, as this is set by a body weight threshold [42]. It is therefore not necessarily the explanation to the unaffected body weight herein, since the crickets were of adult age. The only parameter that affected body weight in this study was sex, in line with female *A. domesticus* generally being heavier than males [4].

### Correlations between immune parameters and protein content

Positive correlations between PO activity and haemocyte count were found in animals reared in 16L:8D, although tendencies were seen in all crickets and in the group of *ad libitum* fed crickets. This is also in line with previous findings [8,29]. It is worth noting that fasting had a main effect on both PO activity and haemocyte count, but a correlation was not found in the fasted group of crickets, indicating that there could be a difference in how sensitive these parameters are towards 48 h of fasting.

Positive correlations between haemocyte count and protein concentration were found in all crickets as well as several groups (females, fasted animals, and in both fasted females (16L:8D) and control females (12L:12D)). The strong correlation in females in general, and in the two female groups, indicates that the correlations seen for all animals and fasted crickets were driven by females. In previous studies, both haemocyte count and protein content have by themselves been linked to an increased chance of survival during an immune challenge [8,27], and our results suggest that these measures are correlated, especially in females. Protein content was also found to be positively correlated with PO activity, in contrast to results in *G. texensis* [27]. PO is a protein within the haemolymph and is therefore also measured in total protein content and could, in theory, be why these parameters are correlated. However, it is unlikely that an increase in PO alone would cause an increase in total protein concentration, since this is just one protein of many in the haemolymph. However, it is possible that other proteins involved in immunity, in addition to PO, are increased if the animal's immune state is good, thereby affecting total protein content. Hence, it is more likely that there is a correlation between immunity proteins and total protein content. This could also explain the correlation between protein content and haemocyte count; an increased investment in immunocompetence of the animal.

In conclusion, protein content in haemolymph was positively correlated to both PO and haemocyte count, suggesting that it might be useful as an indicative measurement of these immune parameters in adult *A. domesticus*.

### General discussion and conclusion

Data on the effect of photoperiod on development in combination with immune parameters are scarce. One study showed that individuals of *G. bimaculatus* that developed faster, grew smaller in size but had more haemocytes and increased PO activity, suggesting that development time and body size affect immune parameters, although this particular study did not find a main effect of photoperiod per se [8]. Another study found that, under constant light, haemocyte count and lytic activity were negatively affected, but PO activity remained unaffected in *T. commodus* [17]. Photoperiod is likely to affect insects differently depending on reproductive season [[5], [6], [7],^9^,^10^]. Some species of multivoltine crickets, e.g. *G. bimaculatus* and *A. domesticus*, might be less affected since their reproductive period is not time constrained [5,8]. As discussed by [8], it is also possible that the effect of the environment, in this case photoperiod, will affect developmental time differently depending on other rearing factors, such as temperature and/or food availability. That is, in a warm enough environment, the minimum developmental time is reached, and no additional effects will be seen when altering e.g. photoperiod. This could possibly explain why no effect of photoperiod was seen on body weight herein. However, main effects of photoperiod for both lysozyme and protein concentrations were found*.* Although the lysozyme data should be interpreted with caution given the low *n*, it is notable that longer days (16L:8D) increased lysozyme concentration and decreased protein concentration, indicating that when housed during longer days, the haemolymph protein has a greater proportion of lysozyme. Photoperiod affected the number of haemocytes differently in males and females, with decreased cell number in females in the 16L:8D versus 12L:12D photoperiod, while males were unaffected. Together, these results imply that photoperiod may be of importance for immunocompetence but needs further investigation.

Immunoredistribution is a process where resources within the immune system relocate to other parts of the body and/or other parameters of the immune system to tackle different obstacles [43,44]. Measuring immunocompetence is therefore complicated, where an insect's immunocompetence is affected by the physiological state, e.g. stress induced by fasting [24,25], which in turn might affect both immunoredistribution and disease resistance. We did not investigate direct resistance to pathogens, which limits conclusions regarding disease resistance. However, immunocompetence may be negatively affected by fasting, and to some extent by photoperiod, which warrants further investigations.

Moreover, age is known to be important for the immune parameters investigated herein, including the number of circulating haemocytes [28,[45], [46], [47]], and a majority of the crickets were ≥ 4 weeks postmoult. Our results revealed large variation in the haemocyte count within most of the groups, possibly due to age since the exact adult age (post moult) of each individual cricket was unknown. Circulating haemocytes decrease with age in e.g. *Gryllus assimilis* [47] and mosquitos (*Aedes aegypti* [45]), but not in *G. texensis* [28]. Results from *A. domesticus* point toward no change in haemocyte count during the first three weeks after adult emergence [35], although the authors discuss that there was a great variability between crickets within the same age. In a study of immunosenescence in *Drosophila melanogaster*, an age-related decrease in haemocyte count was only present in females [46], suggesting a sex-specific decline, which might explain the difference between sexes found in our study. Age could possibly also explain the very low amount of lysozyme, with a more pronounced decline in males. It is therefore possible that more distinct effects would have been seen if the crickets had been younger, around 1–2 weeks post moult, and at a more similar age. However, the present study − where hatching date and adult maturity of the majority of the cohort was registered, not individual maturation − was considered applicable to the cricket farm situation, where crickets are harvested when the majority in a box has reached maturity.

In conclusion, aspects of the immune system in adult *A. domesticus* are affected by different photoperiods and 48 h of fasting. Besides starvation being a competent stressor that may compromise animal welfare per se, our findings may also have implications for immunocompetence and disease resistance. Further studies are needed to confirm these results on a larger population, on an age-specified group to remove the possible effect of age, as well as adding experiments to measure disease resistance. However, the results do show that rearing practices could have important implications for maintaining good immune function in *A. domesticus*.

## Ethics approval

Not applicable.

### Data and model availability statement

Data supporting the conclusions of this article are accessible through the online version of this article.

## Declaration of generative AI and AI-assisted technologies in the writing process

Not applicable.

## CRediT authorship contribution statement

**Frida A. Lindberg:** Writing – review & editing, Writing – original draft, Visualization, Investigation, Formal analysis, Data curation. **Ida Waern:** Writing – review & editing, Resources, Investigation, Funding acquisition. **Emma Nilsson:** Writing – review & editing, Investigation. **Anna Jansson:** Writing – review & editing, Resources. **Lena Holm:** Writing – review & editing, Supervision, Resources, Methodology, Conceptualization. **Erika Roman:** Writing – review & editing, Supervision, Methodology, Funding acquisition, Conceptualization.

## Declaration of competing interest

The authors declare that they have no known competing financial interests or personal relationships that could have appeared to influence the work reported in this paper.

## Data Availability

Data generated in our study are available in the Supplementary material.

## Appendix Supplementary materials

Image, application 1

Image, application 2
