# Supplementary material for: Effect of fasting and two different photoperiods on immune parameters in adult male and female house crickets (Acheta domesticus)
Source: Comp Immunol Rep. 2025 Feb 7;8:200210. doi: 10.1016/j.cirep.2025.200210 (PMC11874521; doi:10.1016/j.cirep.2025.200210)
Supplement: Supplementary file 2 [file mmc2.docx]

**Effect of fasting and two different photoperiods on immune parameters in adult male and female house crickets (*Acheta domesticus*)**

F. A. Lindberg, I. Waern, E. Nilsson, A. Jansson, L. Holm^#^, E. Roman^#^


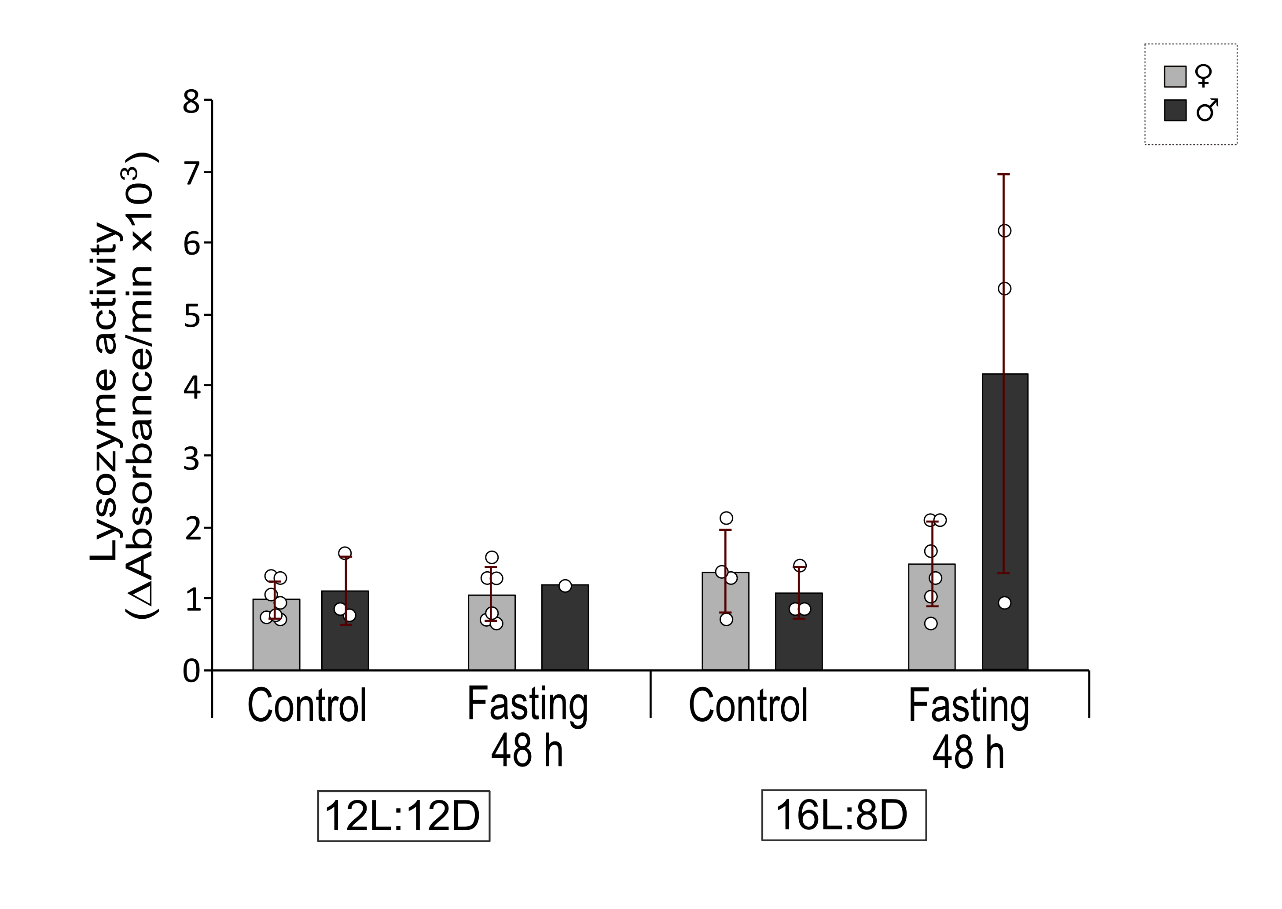


**Fig. S1.** Lysozyme-like activity in haemolymph from adult Acheta domesticus. Only crickets that reached the threshold of the lowest concentration in the standard curve were analysed. Bars denote average values with standard deviation. Each white point corresponds to one individual.12L:12D, 12 hours of light and 12 hours of dark; 16L:8D, 16 hours of light and 8 hours of dark.

**Table S1.** Number of individuals that reached the threshold in the lysozyme assay in the different groups. The threshold was set to the lowest concentration in the standard curve. “Yes” denotes that the threshold was reached, “No” that it was not. Analysis was made with Fisher exact test and a significance level of *p < 0.05* was used.

|  | Number of individuals | Threshold reached | Group |
| --- | --- | --- | --- |
| Sex | | | |
|  | 24 | Yes | Female |
|  | 3 | No | Female |
|  | 10 | Yes | Male |
|  | 16 | No | Male |
|  |  | *P < 0.001* | |
| Light program | | | |
| ♀+♂ | 16 | Yes | 16:8 |
|  | 8 | No | 16:8 |
|  | 18 | Yes | 12:12 |
|  | 11 | No | 12:12 |
|  |  | *P = 0.780* | |
| ♀ | 10 | Yes | 16:8 |
|  | 1 | No | 16:8 |
|  | 14 | Yes | 12:12 |
|  | 2 | No | 12:12 |
|  |  | *P = 1.000* | |
| ♂ | 6 | Yes | 16:8 |
|  | 7 | No | 16:8 |
|  | 4 | Yes | 12:12 |
|  | 9 | No | 12:12 |
|  |  | *P = 0.688* | |
| Treatment | | | |
| ♀+♂ | 16 | Yes | Fasting |
|  | 11 | No | Fasting |
|  | 18 | Yes | Control |
|  | 8 | No | Control |
|  |  | *P = 0.569* | |
| ♀ | 12 | Yes | Fasting |
|  | 2 | No | Fasting |
|  | 12 | Yes | Control |
|  | 1 | No | Control |
|  |  | *P = 1.000* | |
| ♂ | 4 | Yes | Fasting |
|  | 9 | No | Fasting |
|  | 6 | Yes | Control |
|  | 7 | No | Control |
|  |  | *P = 0.688* | |

12:12, 12 hours of light and 12 hours of dark; 16:8, 16 hours of light and 8 hours of dark. Fasting, no feed for 48 hours; Control, fed *ad libitum*.

**Table S2.** List of significant correlations found between the parameters measured.

| **Correlation** | **Group** | **Spearman R** | ***p-value*** |
| --- | --- | --- | --- |
| Protein concentration and PO | All | 0.31 | *0.022* |
|  | Females | 0.56 | *0.001* |
|  | 12L:12D | 0.39 | *0.036* |
| Protein concentration and Haemocyte count | All | 0.38 | *0.006* |
|  | Females | 0.84 | *< 0.001* |
|  | Fasted | 0.42 | *0.026* |
|  | Females, fasted, 16L:8D | 0.83 | *0.042* |
|  | Females, control, 12L:12D | 0.87 | *0.010* |
| PO and Haemocyte count | 16L:8D | 0.42 | *0.042* |

PO, phenoloxidase; 12L:12D, 12 hours of light and 12 hours of dark; 16L:8D, 16 hours of light and 8 hours of dark. Fasted, no feed for 48 hours; Control, fed *ad libitum*.
